# Correction: Luminescence properties of the pink emitting persistent phosphor Pr^3+^-doped La_3_GaGe_5_O_16_

**DOI:** 10.1039/c8ra90024h

**Published:** 2018-03-21

**Authors:** Shaoan Zhang, Yihua Hu, Li Chen, GuiFang Ju, Tao Wang, Zhonghua Wang

**Affiliations:** School of Physics and Optoelectronic Engineering, Guangdong University of Technology Waihuan Xi Road, No.100 Guangzhou 510006 People’s Republic of China huyh@gdut.edu.cn +86-20-39322265 +86-20-39322262

## Abstract

Correction for ‘Luminescence properties of the pink emitting persistent phosphor Pr^3+^-doped La_3_GaGe_5_O_16_’ by Shaoan Zhang *et al.*, *RSC Adv.*, 2015, **5**, 37172–37179.

The authors regret that Fig. 2a in the original manuscript shows an incorrect image. The correct Fig. 2a is shown below.

**Fig. 2:**
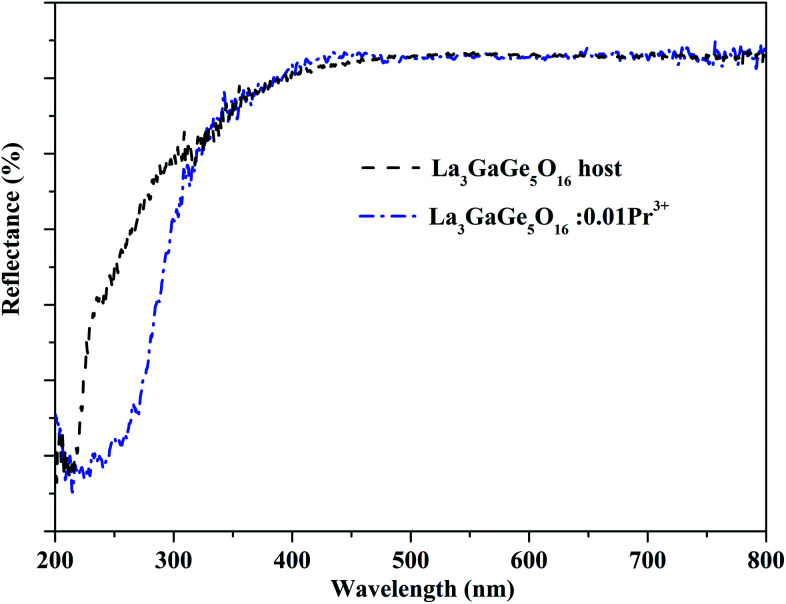
(a) Diffuse reflectance spectra of pure La_3_GaGe_5_O_16_ and La_3_GaGe_5_O_16_:0.01Pr^3+^ at room temperature.

The Royal Society of Chemistry apologises for these errors and any consequent inconvenience to authors and readers.

## Supplementary Material

